# Suitability of IS*6110*-RFLP and MIRU-VNTR for Differentiating Spoligotyped Drug-Resistant *Mycobacterium tuberculosis* Clinical Isolates from Sichuan in China

**DOI:** 10.1155/2014/763204

**Published:** 2014-03-03

**Authors:** Chao Zheng, Yuding Zhao, Guoqiang Zhu, Song Li, Honghu Sun, Qin Feng, Mei Luo, Fanzi Wu, Xuefeng Li, Véronique Hill, Nalin Rastogi, Qun Sun

**Affiliations:** ^1^Key Laboratory of Bio-Resources and Eco-Environment of the Ministry of Education, College of Life Sciences, Sichuan University, Chengdu, Sichuan 610065, China; ^2^Chengdu Longquan Health School, Chengdu, Sichuan 610100, China; ^3^Chengdu Anti-Tuberculosis Hospital, Chengdu, Sichuan 610016, China; ^4^WHO Supranational TB Reference Laboratory, Institut Pasteur de Guadeloupe, BP 484, 97183 Abymes Cedex, France

## Abstract

Genotypes of *Mycobacterium tuberculosis* complex (MTBC) vary with the geographic origin of the patients and can affect tuberculosis (TB) transmission. This study was aimed to further differentiate spoligotype-defined clusters of drug-resistant MTBC clinical isolates split in Beijing (*n* = 190) versus non-Beijing isolates (*n* = 84) from Sichuan region, the second high-burden province in China, by IS*6110*-restriction fragment length polymorphism (RFLP) and 24-locus MIRU-VNTRs. Among 274 spoligotyped isolates, the clustering ratio of Beijing family was 5.3% by 24-locus MIRU-VNTRs versus 2.1% by IS*6110*-RFLP, while none of the non-Beijing isolates were clustered by 24-locus MIRU-VNTRs versus 9.5% by IS*6110*-RFLP. Hence, neither the 24-locus MIRU-VNTR was sufficient enough to fully discriminate the Beijing family, nor the IS*6110*-RFLP for the non-Beijing isolates. A region adjusted scheme combining 12 highly discriminatory VNTR loci with IS*6110*-RFLP was a better alternative for typing Beijing strains in Sichuan than 24-locus MIRU-VNTRs alone. IS*6110*-RFLP was for the first time introduced to systematically genotype MTBC in Sichuan and we conclude that the region-adjusted scheme of 12 highly discriminative VNTRs might be a suitable alternative to 24-locus MIRU-VNTR scheme for non-Beijing strains, while the clusters of the Beijing isolates should be further subtyped using IS*6110*-RFLP for optimal discrimination.

## 1. Introduction

Tuberculosis (TB) remains a serious public health issue and a leading cause of adult mortality arising from a single infectious agent. China is not only the top-two country with respect to the estimated number of cases and deaths but also a hotspot of drug resistance [1]. Sichuan, located in southwestern China, has the second-greatest number of TB cases of any Chinese province. The prevalence of drug-resistant TB, especially multidrug-resistant (MDR) TB, is much higher than average for a developed region in eastern China [2–4]. An official report in 2008 revealed that MDR-TB in Sichuan was found in the sputum of 28.3% of all smear-positive, and the proportion of strains showing resistance to at least one drug was 74% [5], a figure maintained at 70% in 2013 [6]. In such a context, investigations on genetic diversity of drug resistant *M. tuberculosis* clinical isolates may provide with useful information about the origin and transmission of the circulating isolates [7–10].

Although initially considered as the gold standard for identifying epidemiologically linked isolates from patients, the IS*6110*-restriction fragment length polymorphism (IS*6110*-RFLP) was time-consuming and expensive and characterized by disadvantages such as requirement of good quality DNA, the presence of strains harboring low/no copy number of IS*6110* element in many parts of Asia and certain parts of the world, and difficulties in inter-laboratory comparison of the IS*6110*-RFLP patterns [11–14]. Subsequently, it was later replaced by PCR-based rapid methods such as mycobacterial interspersed repetitive units-variable number of DNA tandem repeats (MIRU-VNTRs) typing and spoligotyping [15]. Although spoligotyping can identify Beijing family isolates easily [16], classification of *M. tuberculosis* strains into robust phylogenetic lineages is not always possible and misclassifications may occur occasionally [17, 18]. Considered one of the most successful *M. tuberculosis* lineages involved in tuberculosis transmission [19], the spoligotyping-defined Beijing clusters are subtyped using more discriminatory tools such as IS*6110*-RFLP and MIRU-VNTRs to avoid overestimating the clustering rate; and VNTR typing is usually preferred to RFLP on genotyping *M. tuberculosis* in regions where Beijing genotype isolates make up a low percentage of TB population [20]. However, as was shown recently in China [21], Russia [22], and Japan [23], the 24-locus MIRU-VNTR scheme is not always sufficient for discriminating Beijing isolates in regions with a high prevalence of Beijing strains, leading to the development of region-adjusted complementary typing schemes [22–24], and more recently proposal of a consensus set of Hypervariable MIRU-VNTRs for subtyping Beijing isolates [25].

There is an increasing amount of evidence regarding the importance of subtle regional differences in Beijing genotypes due to evolutionary aspects, for example, Billamas et al. reported that in Thailand, the variations of one or more MIRU-VNTR loci in Beijing isolates of highly similar IS*6110*-RFLP patterns could imply the occurrence of evolutionary MIRU-VNTR loci among genetically homogeneous *M. tuberculosis* clinical isolates [26]. Previous studies demonstrated that *M. tuberculosis* population structure varied geographically in China, mainly from north to south, with southern China showing a relatively smaller proportion of Beijing isolates with a greater distribution frequency of non-Beijing types [27, 28]. Our concomitant research focused on a detailed analysis of spoligotyping-based patterns (HGI = 0.595), underlying a more diverse population structure of drug-resistant *M. tuberculosis* isolates in Sichuan than in other parts of China [13]. Observed data suggest that subtle differences in *M. tuberculosis* transmission and epidemiology may exist in Sichuan, making the overall population structure observed quite different from that observed in northern China.

In such a context, the objective of the present study was to further differentiate the spoligotyped drug-resistant *M. tuberculosis* clinical isolates circulating in Sichuan by IS*6110*-RFLP and MIRU-VNTRs, so as to develop appropriate genotyping methodology for various *M. tuberculosis* lineages, particularly the Beijing versus non-Beijing family isolates.

## 2. Materials and Methods

### 2.1. Mycobacterial Isolates

A total of 306 drug-resistant *Mycobacterium tuberculosis* clinical isolates were randomly selected from isolates showing any sort of resistance. All strains were cultured from samples collected at the Chengdu Antituberculosis Hospital, the only professional antituberculosis hospital in Sichuan, from January 2008 to August 2009. Clinical data were obtained from the subjects' medical records without any invasion of patients' privacy, and the results of this study did not influence patient's treatment in any way.

### 2.2. Drug Susceptibility

Specimens were collected and disposed in accordance with WHO guidelines. Briefly, strains were cultured on Löwenstein Jensen (LJ) slants at 37°C and MTBC isolates were identified using standard biochemical methods such as susceptibility to p-nitrobenzoic acid (PNB) and to 2-thiopnene carboxylic acid hydrazide (TCH), pyrazinamidase activity (PZA), nitrate reduction, and niacin production. Drug susceptibility testing was done using proportion method with streptomycin (STR), 10 mg/mL; isoniazid (INH), 0.2 mg/mL, rifampin (RIF), 40 mg/mL and ethambutol (EMB), 2 mg/mL.

### 2.3. DNA Extract and Genotyping

Genomic DNA from clinical isolates was extracted using the CTAB method [29] followed by IS*6110*-RFLP according to an international protocol [30]. Briefly, genomic DNA was extracted, digested with *Pvu*II, and subjected to agarose gel electrophoresis. After DNA was blotted to a Hybond membrane, DNA fingerprinting was performed using hybridization with the IS*6110* insertion sequence and enhanced chemiluminescence assay (Roche). The spoligotyping and MIRU-VNTR information of all drug-resistant isolates were from our previous study [13]. Spoligotyping was performed as described by Kamerbeek et al. [10]. For MIRU-VNTRs, all 24 loci were amplified with corresponding primers as described by Supply et al. [31].

### 2.4. Database Comparison

Both the spoligotypes and MIRU-VNTR patterns were compared using the SITVIT2 proprietary database of Institut Pasteur de la Guadeloupe, which is an updated version of the previously released SpolDB4 [32] and SITVITWEB databases [33] (available online at http://www.pasteur-guadeloupe.fr:8081/SITVIT_ONLINE/). In this database, spoligotype international type (SIT) and MIRU international type (MIT) designate spoligotype and MIRU patterns shared by 2 or more patient isolates, as opposed to “orphan” which designates patterns reported for a single isolate. The major phylogenetic clades were assigned according to the signatures provided in the database, which defined 62 genetic lineages/sublineages in SpolDB4, with 5 “new rules” for the definition of variants within existing lineages in SITVITWEB and SITVIT2 [32, 33]. These include various *M. tuberculosis* complex members such as *M. bovis*, *M. caprae*, *M. microti*, *M. canetti*, *M. pinipedi*, and *M. africanum*, as well as rules defining major lineages/sublineages for *M. tuberculosis* sensu stricto. These include the Beijing clade, the Central Asian (CAS) clade and 2 sublineages, the East African-Indian (EAI) clade and 9 sublineages, the Haarlem (H) clade and 3 sublineages, the Latin American-Mediterranean (LAM) clade and 12 sublineages, the ancestral “Manu” family and 3 sublineages, the S clade, the IS*6110*-low-banding X clade and 3 sublineages, and an ill-defined T clade with 5 sublineages.

### 2.5. Statistical Analysis

The analysis of the size of PCR fragments and assignment of the various VNTR alleles were achieved using Quantity One (version 4.6.2) software (Bio-Rad Laboratories). The Hunter-Gaston index (HGI) was calculated as described previously [34]. It was used to evaluate the level of discriminatory power of the typing methods and the allelic diversity of individual VNTR loci. Fingerprints of IS*6110* were analyzed using BioNumerics (version 6.1) software (Applied Maths, Kortrijk, Belgium). Similarities between RFLP patterns were calculated using the Dice coefficient, and the dendrogram was produced using an unweighted pair group method with an arithmetic averages algorithm.

## 3. Results

### 3.1. Subclustering Beijing and Non-Beijing Isolates by IS*6110*-RFLP

**Figure 1:**
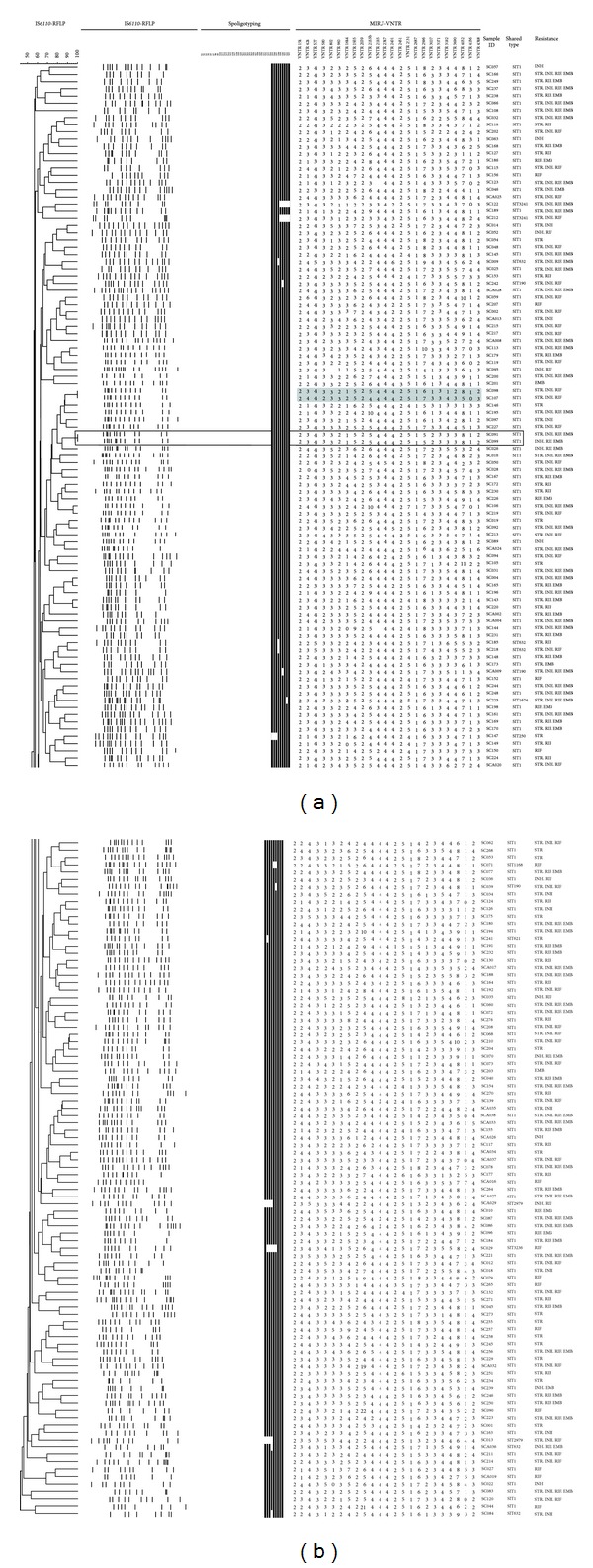
IS*6110*-RFLP based dendrogram of 190 clinical isolates of Beijing family. Gray highlighting indicates isolates with identical IS*6110* patterns differentiated by 24-locus MIRU-VNTR. Isolates in blank boxes are fully identical genotypes.

**Figure 2:**
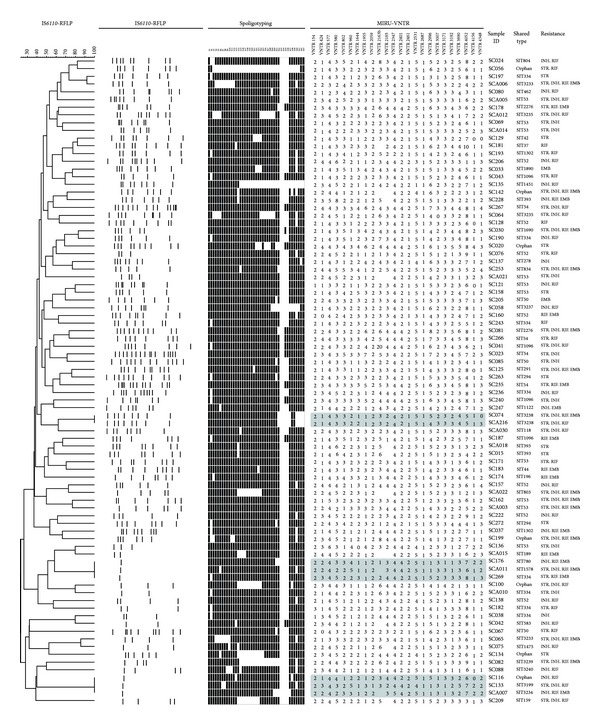
IS*6110*-RFLP based dendrogram of 84 clinical isolates of non-Beijing family. Gray highlighting indicates isolates with identical IS*6110* patterns. These were differentiated using 24-locus MIRU-VNTR.

A subset of 274 isolates with sufficient DNA was available for IS*6110*-RFLP analysis. The 274 isolates were divided into two groups (Beijing and non-Beijing family) based on spoligotyping results after checking them against the SITVIT2 database. Each group was subjected to IS*6110*-RFLP analysis. Of the 274 isolates, six clusters and 260 unique patterns were identified using IS*6110*-RFLP (HGI = 0.999) (Table 1). One of these clusters was split by spoligotyping. The Beijing family strains (*n* = 190; 69.34%) were subdivided into two clusters and 185 unique patterns (Figure 1), while the non-Beijing family strains (*n* = 84; 30.66%) were subdivided into three clusters and 75 unique patterns (Figure 2). The clusters varied in size from two to three isolates.

The numbers of IS*6110* bands ranged from 7 to 17 among the Beijing family and from 1 to 16 among the non-Beijing family (Table 2). One hundred and seventy of the 190 (89.5%) were Beijing family strains with more than 10 copy numbers of IS*6110*, and the isolates with less than 6 copy numbers constituted about 39.3% of all non-Beijing family, including ill-defined T family (60.6%), Manu2 (9%), LAM (3%), and unknown or orphan clade (15.2%, 12.1%). Three of the 51 ill-defined T family (5.9%), two of the 9 unknown clade, and one of 7 orphan clade strains harbored only one copy of IS*6110* element.

### 3.2. IS*6110* Clusters and VNTR Clusters Subdivided by Each Other or Spoligotyping

Among 274 spoligotype-defined isolates, two shared a fully identical genotype, indicating a concordance of the genotyping methods used, that is, spoligotyping, 24-locus MIRU-VNTR and IS*6110*-RFLP. Both isolates belonged to the Beijing family (SIT1) harboring 12 copies of IS*6110*. One RFLP cluster of 2 isolates (SC041 and SC044; both of them had 8 copies of IS*6110*) was split by either spoligotyping or VNTR approach by two loci (QUB-11b, MIRU39). Other two RFLP clusters were further resolved by VNTR method only. One cluster contained 2 isolates of ill-defined T family harboring 8 copies of IS*6110*, and the other cluster contained 2 isolates of Beijing family harboring 12 copies of IS*6110*. Among the non-Beijing family strains, the six isolates harboring only one copy of IS*6110* were grouped into two clusters of 3 isolates each, and they were differentiated by multiple MIRU loci and spoligotyping.

The allelic diversity of individual MIRU-VNTR loci was given in Table 3. The HGI value varied significantly from null to 0.852. The 24-locus MIRU-VNTR typing differentiated 274 strains into 6 clusters containing 12 isolates, and there was only one isolate belonged to non-Beijing family (Manu2 clade). Except the fully identical genotypes, one VNTR cluster was differentiated by both spoligotyping and IS*6110*-RFLP, which differed by 6 RFLP bands. Three out of six VNTR clusters split by RFLP differed by only one RFLP band and another one differed by three RFLP bands. There were 12 highly discriminatory loci (HGI > 0.6) including locus 424, 802, 960, 1644, 1955, 2163b, 2996, 3007, 3192, 3690, 4052, and 4348. Compared with the 24-locus MIRU-VNTR, one more cluster was identified by this 12-locus scheme, which contained 2 isolates (SC169 and SC172) belonging to Beijing family (SIT1). The 12-locus scheme showed a discrimination power equal to that of 24-locus MIRU-VNTR and IS*6110*-RFLP for the studied sample (the HGI values evaluated for different sets of MIRU-VNTR and IS*6110*-RFLP were illustrated in Table 1).

## 4. Discussion

The ongoing transmission of *M. tuberculosis* in certain settings is heavily influenced by the prevailing population structure of tubercle bacilli, and certain genetic families of this species have attracted more attention due to their global dissemination and/or remarkable pathogenic properties [35], such as the predominance of a highly homogeneous group like the Beijing family in northern China, Japan, and South Korea, where its proportions can be as high as 70–92.59% [22, 28, 36]. This differs notably from the predominance of LAM strains in South America and all subregions of Africa (except AFRI being the most predominant in Western Africa), *M. africanum* in West-Africa, Haarlem, X, and T in Europe, and CAS and EAI in the Indian subcontinent [32, 33]. These regional variations in *M. tuberculosis* population structure require that a baseline value of circulating genotypes is established to facilitate typing schemes suitable for each geographical setting. We considered it desirable to evaluate the relative efficiency of the 24-locus MIRU-VNTRs and the IS*6110*-RFLP for differentiating spoligotype-defined clusters of drug-resistant *M. tuberculosis* clinical isolates from Sichuan.

A recent study reported that the Beijing family strains acquired drug resistance *in vitro* more rapidly and were preferentially associated with resistance to multiple drugs than Euro-American lineage [37]. In Sichuan, spoligotyping-based analysis showed that Beijing family represented 69.28% of all isolates and so constituted the largest group (66.24%) of MDR-TB [13]; a finding that required further differentiation of the clinical isolates using genotyping methods of higher discriminatory power. The high variability in copy number and location of IS*6110*, as well as its stability over time, renders IS*6110*-RFLP typing as a useful diagnostic and epidemiological tool [19], but it fails to adequately differentiate *M. tuberculosis* strains with identical patterns of low copy numbers of IS*6110* isolates [11, 14, 38, 39]. As compared to other *M. tuberculosis* strains, the Beijing isolates are characterized by specific IS*6110 *insertion points [19]; in our study 89.5% of Beijing family isolates contained >10 copies of IS*6110*, and the proportion of low-copy-number (≤6) isolates was null, showing an excellent discriminatory power, an observation that was consistent with other studies [40–42].

The discriminatory power of 24-locus MIRU-VNTR was reported to be comparable to that of IS*6110*-RFLP when combined with spoligotyping [43, 44]. Nonetheless, the rate of clustering is not a unique index to compare two typing methods due to the different characteristics involved in case of over-clustering observed by 2 different methods. Thus, even though VNTR typing is certainly useful as a secondary means of typing *M. tuberculosis* with low copy numbers of IS*6110 *[11], it cannot alone define all unique isolates. This is particularly true for discriminating the Beijing genotype strains, and some loci beyond the 24-locus MIRU format have permitted to further subdivide the Beijing clusters in several countries, including Russia, Japan, and Kyrgyzstan [23, 25, 45, 46]. In a comparative 5-year nationwide survey of IS*6110*-RFLP versus 24-locus VNTR typing in the Netherlands [20], the authors showed that although the level of discrimination did not differ substantially among the two methods, the global concordance—defined as isolates labeled unique or identically distributed in clusters by both methods—amounted to only 78.5%. Of the remaining cases, 12% were only clustered by VNTRs, 7.7% only by RFLP, and 1.8% revealed altogether different cluster compositions by the two approaches. A similar observation may exist in our study by comparing these two methods, and apparently there was a slight tendency of over-clustering by 24-locus VNTRs as compared to IS*6110*-RFLP for Beijing strains. All clusters were related to the Beijing family by 24-locus VNTRs (Table 1), while three of the potential VNTR over-clusters were split by RFLP, though the subclusters were apparently closely related since they differed by only one RFLP band.

Among non-Beijing family strains, two isolates sharing identical IS*6110*-RFLP and spoligotypes (SIT3238) profiles and 6 isolates in two clusters with a single IS*6110* copy were completely differentiated by VNTRs. The general experience is that *M. tuberculosis* isolates contain multiple IS*6110* elements and that strains with the same IS*6110* fingerprints are epidemiologically related [39], but many *M. tuberculosis* isolates in South and Southeast Asia, including Thailand and India, have only a few copies or even single copy of IS*6110* [11, 47, 48] making the discriminating power of IS*6110*-RFLP too low to be useful for inferring epidemiological linkages [11]. We too encountered a similar situation; nonetheless almost all the isolates with less than 6 copy numbers of IS*6110* belonged to non-Beijing family in our study. The low-copy-number isolates in our study ranked in the following order: orphan strains, 4/7 or 57.1%; unknown clade, 5/9 or 55.6%; LAM9 1/2 or 50%; ill-defined T family, 20/51 or 39.2%; Manu2, 3/10 or 30%. Three out of 6 isolates harboring only one copy of IS*6110* element belonged to ill-defined T family, which was the second most frequent family (18.6%).

**Figure 3:**
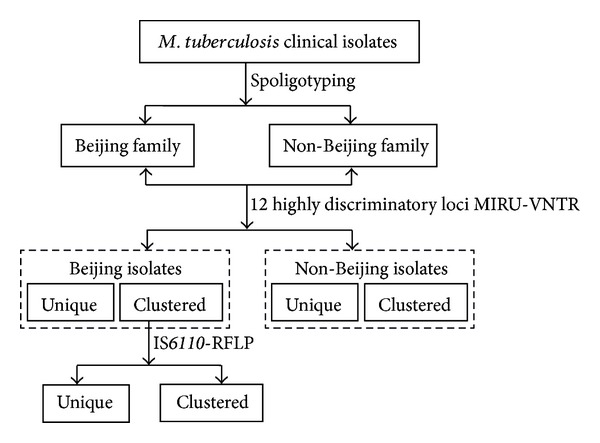
Schematic overview of the typing scheme developed to differentiate *M. tuberculosis* clinical isolates.

However, despite a high clustering rate by IS*6110*-RFLP in many parts of Asia and certain parts of the world [11, 12, 49, 50], the switch to IS*6110*-RFLP typing may present downsides such as overestimating the proportion of clustered *M. tuberculosis* isolates of the non-Beijing family (e.g., the ill-defined T family in Sichuan). Thus 24-locus VNTR typing should be maintained for differentiating *M. tuberculosis* isolates with low copy numbers of IS*6110*. Although the discriminatory power of VNTR typing can be increased by implementing more MIRU-VNTR loci scattered through genome of *M. tuberculosis*, the phylogeographically diverse population structure of *M. tuberculosis* may make first-line typing sets country- and region-specific, facilitating the inclusion of specific MIRU-VNTR loci in different typing schemes [40, 45]. Since both 24-locus MIRU-VNTR and spoligotyping had a tendency to overestimate the proportion of clustered isolates of Beijing family which could be easily overcome by IS*6110*-RFLP; combining 12 highly discriminatory MIRU-VNTR loci with IS*6110*-RFLP could be a suitable alternative to 24-locus scheme for typing Beijing strains in Sichuan (Figure 3). On the other hand, IS*6110*-RFLP does not seem appropriate for differentiating non-Beijing strains due to over-clustering, which are equally and sufficiently discriminated both by 24-locus MIRU-VNTR and 12 highly discriminatory MIRU-VNTR loci (Table 1). In such as a case, the 12 highly discriminatory loci scheme consisting of the most polymorphic loci and requiring less time and labor deems suitable for non-Beijing family strains (Figure 3).

Systematic genotyping may help predict the spreading of MDR strains and improve the management of TB [50]. It has been suggested that isolates with higher IS*6110* copies may evolve increased selective advantages (such as drug resistance, virulence, and efficient transmission) over those with fewer copies [41, 51–53]. Whether studies aiming to infer the potential implication of the IS*6110* elements as underlying mechanisms for successful emergence of Beijing genotype strains would fully answer the genotype and phenotype relationships and explain the current transmission patterns and spread of drug-resistant TB remains debatable [19, 54–56]. In our study, 150 (78.94%) Beijing isolates were found to harbor 10 to 14 IS*6110* copies, 20 harbor 7 to 9 copies, and only 20 harbor 15 to 17 IS*6110* copies (Table 2). This may indicate that Beijing family strains in Sichuan were prone to moderate copies of IS*6110* elements, disclosing a different IS*6110*-RFLP profile distinct from those observed in Beijing and Myanmar [23, 43]. On average, the W-Beijing family possesses a higher number of IS*6110* copies (around 21) than any other lineage [41]. In our study, multidrug-resistance was observed among all non-Beijing isolates [29]. Five out of 6 non-Beijing isolates with only one IS*6110* copy were MDR-TB (Figure 2). These results may support the fact that low-copy-number strains that belonging primarily to the non-Beijing family also evolve selective advantages and cause the transmission of drug-resistance and even outbreaks [51, 52].

## 5. Conclusion

This is the first investigation to differentiate *M. tuberculosis* isolates from Sichuan province by IS*6110*-RFLP. The results obtained showed that combining 12 highly discriminatory MIRU-VNTR loci with IS*6110*-RFLP was a better alternative for typing Beijing strains in Sichuan than 24-locus MIRU-VNTRs alone. Furthermore, the 12 highly discriminative loci scheme developed in this study showed a resolution equal to that of IS*6110*-RFLP and 24-locus MIRN-VNTR for discrimination of non-Beijing strains.

## Figures and Tables

**Table 1:** Discriminatory power of the three different typing methods for a subset of 274 drug-resistant* M. tuberculosis* isolates from Sichuan, China.

Method	Number of types	Number of unique isolates	Number of clustered isolates	Number of clusters	Range of cluster size (number of isolates)	Number of clustered Beijing isolates	Number of clustered non-Beijing isolates	HGI
24-locus MIRU-VNTR	268	262	12	6	2	11	1	0.999
12 highly discriminatory loci	267	260	14	7	2	13	1	0.999
IS*6110*-RFLP	266	260	14	6	2-3	5	9	0.999

**Table 2:** IS*6110*-RFLP analysis of Beijing and non-Beijing isolates from Sichuan.

Number of IS*6110* copies	Number of isolates	
	Beijing isolates (*n* = 190)	Non-Beijing isolates (*n* = 84)
1	0	6
2–6	0	27
7–9	20	39
10–14	150	11
15–17	20	1

**Table 3:** Allelic diversity of the 24 MIRU-VNTRs loci in 274 drug-resistant *M. tuberculosis* isolates from Sichuan, China.

Schemes of VNTR locus	VNTR locus	VNTR alias	No. of alleles	Range of repeats	Allelic diversity (HGI) for		
					Beijing isolates (*n* = 190)	Non-Beijing isolates (*n* = 84)	All isolates (*n* = 274)
Discriminatory loci	424	Mtub04	6	0–4, 6	**0.722**	**0.705**	**0.745**
	577	ETR-C	5	2–6	0.111	0.159	0.126
	580	MIRU4	7	1–6, 8	0.185	**0.65**	0.367
	802	MIRU40	5	1–5	0.558	**0.727**	**0.643**
	960	MIRU10	6	0–5	0.589	0.596	**0.627**
	1644	MIRU16	6	0–5	**0.664**	**0.689**	**0.673**
	1955	Mtub21	9	1–9	**0.706**	**0.73**	**0.803**
	2163b	QUB-11b	15	0–10, 19–22	**0.783**	**0.852**	**0.829**
	2165	ETR-A	5	1–5	0.262	**0.678**	0.502
	2401	Mtub30	5	2–6	0.140	0.265	0.438
	2996	MIRU26	10	1–10	**0.727**	**0.724**	**0.79**
	3192	MIRU31	6	1–6	0.586	**0.697**	**0.708**
	3690	Mtub39	6	1–6	**0.626**	**0.792**	**0.708**
	4052	QUB-26	11	1–11	**0.759**	**0.828**	**0.782**
	4156	QUB-4156	7	0–7	**0.605**	0.479	0.571
Additional loci	154	MIRU2	3	1–3	0	0.07	0.022
	2059	MIRU20	2	1-2	0.031	0.069	0.043
	2347	Mtub29	4	2–5	0.052	0.093	0.064
	2461	ETR-B	3	1–3	0.031	0.488	0.236
	2531	MIRU23	7	1, 3–8	0.176	0.199	0.183
	2687	MIRU24	1	1	0	0	0
	3007	MIRU27	5	0–4	0.599	**0.662**	**0.624**
	3171	Mtub34	4	1–4	0.120	0.137	0.125
	4348	MIRU39	6	1–6	**0.734**	**0.696**	**0.751**

In bold: the HGI > 0.6.

The 12-loci-VNTR scheme was developed based on the highly discriminatory loci (HGI > 0.6) for all isolates. Both discriminatory and additional loci were from Supply et al. [31].
